# Metabolic syndrome and adipokine levels in systemic lupus erythematosus and systemic sclerosis

**DOI:** 10.1007/s10067-021-05731-6

**Published:** 2021-04-11

**Authors:** Antonietta Gigante, Francesco Iannazzo, Luca Navarini, Maria Chiara Sgariglia, Domenico Paolo Emanuele Margiotta, Valentina Vaiarello, Federica Foti, Antonella Afeltra, Rosario Cianci, Edoardo Rosato

**Affiliations:** 1 Department of Translational and Precision Medicine, Sapienza University of Rome, Rome, Italy; 2 Unit of Allergology, Clinical Immunology and Rheumatology, Department of Medicine, Campus Bio-Medico University, Rome, Italy

**Keywords:** Adipokines, Adiponectin, Metabolic syndrome, Resistin, Systemic lupus erythematosus, Systemic sclerosis

## Abstract

**Introduction:**

Aims of study were to evaluate the prevalence of metabolic syndrome (MetS) in systemic lupus erythematosus (SLE) and systemic sclerosis (SSc) patients and to evaluate serum level of adipokines in SLE and SSc patients with and without MetS.

**Methods:**

Fifty SLE patients and 85 SSc patients were enrolled. The diagnosis of MetS was made according to the criteria of the National Cholesterol Education Program (NCEP) Adult Treatment Panel III. Clinical assessment and serum levels of adiponectin and resistin were evaluate in SLE and SSc patients.

**Results:**

Prevalence of MetS was significantly (*p*<0.0001) higher in SLE patients than SSc patients (36% vs 10.6%). Median values of resistin were significantly (*p*<0.001) higher in SLE patients with MetS than SLE patients without MetS [4.01 ng/mL (2.7–4.5) vs 1.92 ng/mL (1.2–3)]. Median values of adiponectin were significantly (*p*<0.05) lower in SLE patients with MetS than SLE patients without MetS [5.64 ng/mL (4.96–8) vs 8.38 ng/mL (6.54–11.01)]. Systemic Lupus Erythematosus Activity Index [8 (6–12) vs 10 (6–13), *p*<0.01] and Systemic Damage Index [2 (1–3) vs 2 (0–3), *p*<0.001] were significantly higher in MetS patients than in patients without MetS.

In SSc, the median value of disease severity scale was significantly higher (*p*<0.05) in MetS patients than in patients without MetS [7 (5–7) vs 5 (3–6)].

**Conclusion:**

Prevalence of MetS is higher in SLE patients. In SLE patients, MetS showed an association with adipokine levels and inflammation/activity disease scores. In SSc patients, MetS was associated with severity of disease.

**Table:** 

**Key Points** *• Prevalence of metabolic syndrome is higher in SLE patients than SSc patients.* *• Resistin is higher in SLE patients with metabolic syndrome.* *• Adineponectin is lower in SLE patients with metabolic syndrome.* *• Disease severity scale is higher in SSc patients with metabolic syndrome.*

## Introduction

The metabolic syndrome (MetS) is characterized by visceral obesity, insulin resistance, hypertriglyceridemia, low high-density lipoproteins (HDL), and arterial hypertension [1]. In the general population, MetS is a predictor marker for the development of cardiovascular (CV) events [2]. The prevalence of MetS is age-related and depends by race and ethnicity. In USA, MetS is reported in approximately 34% of people studied according to National Cholesterol Education Program (NCEP/ATPIII) revised criteria [3]. A similar prevalence was reported in the Iranian population (32.1%) and Mexican population (36%) [4, 5]. In Europe, China, and Middle-East Country, the Mets prevalence is 25% [6–8].

In systemic lupus erythematosus (SLE) patients, the risk of CV events is increased [9]. SLE is an autoimmune disease characterized by autoantibodies production and deposition of immune complexes in several organs [10]. Premature atherosclerosis is higher in SLE patients than general population [11]. The prevalence of MetS in SLE ranges from 18 to 30% [12, 13].

Systemic sclerosis (SSc) is an autoimmune disease characterized by endothelial dysfunction, dysregulation of immune system, and fibrosis of the skin and internal organ [14]. Many complications of SSc are due to endothelial damage. In SSc patients, the prevalence of MetS is 36% and it was associated with CV events [15, 16].

Adipokines (leptin, adiponectin, and resistin) are a heterogenic group of molecules closely associated with MetS [17]. Adiponectin exhibits anti-fibrotic and anti-inflammatory properties. The dysregulation of the cytokines and adipokines is a common feature in SLE and SSc patients [18, 19].

The aim of the study is to evaluate the prevalence of MetS in SLE and SSc patients and to evaluate correlations of MetS with the clinical variables of diseases. The secondary aim is to evaluate serum level of adipokines in SLE and SSc patients with and without MetS.

## Materials and methods

### Study population

Fifty consecutive SLE fulfilled the Hochberg-modified American College of Rheumatology (ACR) classification criteria [20] and 85 consecutive SSc patients fulfilled the 2013 ACR/European League Against Rheumatism criteria [21] were enrolled in this study. All patients were selected by single Centre (Internal Medicine Unit of Sapienza University of Rome-Policlinico Umberto 1).

Exclusion criteria were recent pregnancy, breakfasting, active malignancy, renal failure, secondary hypertension, therapy at an equivalent dose of prednisone ≥ 10 mg/day, and mood disorders.

The study complies with the Declaration of Helsinki. The local ethical committee approved the research protocol (5435) and informed consent was obtained from all patients.

### Assessment of metabolic syndrome

The diagnosis of MetS was made according to the criteria of the National Cholesterol Education Program (NCEP) Adult Treatment Panel III (ATP III) [22]. In adults, MetS is defined by the NCEP/ATP III as having 3 or more of the following: waist in men≥102 cm and in women≥88 cm, high circulating triglycerides ≥150 mg/dL, low levels of high-density lipoprotein<40 mg/dL for men and <50 mg/dL for women, high fasting blood glucose ≥100 mg/dL, and diagnosis of arterial hypertension.

### Clinical assessment

In SLE patients, disease activity was assessed by the Systemic Lupus Erythematosus Activity Index 2000 (SLEDAI-2K) and disease damage by Systemic Lupus International Collaborating Clinics/ACR Damage Index (SDI) [23, 24].

SSc patients were grouped in limited cutaneous (lcSSc) and diffuse cutaneous (dcSSc) according to LeRoy [25]. The modified Rodnan skin score (mRss) was used to evaluate the skin thickening [26]. Disease activity and disease severity were assessed by disease activity index (DAI) and disease severity scale (DSS) [27, 28].

### Nailfold videocapillaroscopy

Nailfold videocapillaroscopy (NVC) was performed with a videocapillaroscope (Pinnacle Studio Version 8) equipped with a ×500 optical probe. According to Cutolo et al., the “SSc pattern” was classified as early, active and late [29].

### Adipokines

Serum levels of resistin and adiponectin, expressed as ng/mL, were measured by commercial ELISA kits (AdipoGen Inc., Seoul, Korea). Blood samples were collected after 12 h of overnight fasting.

### Statistical analysis

All results are expressed as means ± SD or median and interquartile range (IQR). SPSS version 25.0 software was used for statistical analysis. The Shapiro–Wilk test was used to evaluate normal data distribution. Group comparisons were made by Student’s unpaired 2-tailed *t*-test or Mann-Whitney test. Pearson product-moment correlation coefficient or Spearman’s rank correlation coefficient was used to test for associations between numerical variables. The chi-square test or Fisher’s exact test was used to compare categorical variables. *P*-values < 0.05 were considered significant.

## Results

Fifty Caucasian SLE patients [50 F, median age 38 years (36–49)] and 85 Caucasian SSc patients [50 F, median age 57 years (46–65)] were enrolled in this study. Table 1 shows patients’ epidemiological and clinical features. Median age of SLE patients was significantly lower (*p*<0.0001) than SSc patients [38 years (36–49) vs 57 years (46–65)]. Prevalence of MetS was significantly (*p*<0.0001) higher in SLE patients than SSc patients (36% vs 10.6%). Also other criteria of NCEP/ATP III for MetS, except for waist, were significantly more frequent or higher in SLE patients than SSc patients (Table 1).

**Table 1:** Patients’ epidemiological and clinical features

	SSc	SLE	*p* value
Female, *n* (%)	74 (87.1)	50 (100)	0.007
Age, years	58 (46–65)	38 (36–49)	<0.0001
Disease duration, years	11 (7–16)	7 (4–12)	0.03
BMI, Kg/m^2^	23.2 (21.2–24.6)	24.2 (22–28)	0.008
MetS, *n* (%)	9 (10.6)	18 (36)	<0.0001
Waist circumference (cm)	81 (74–89)	79 (71–96)	>0.05
Total cholesterol (mg/dL)	191 (161–212)	201 (181–220)	<0.0001
HDL (mg/dL)	62 (50–77)	65 (56–76)	<0.0001
LDL (mg/dL)	92 (79–120)	112 (97–135)	<0.0001
Triglycerides (mg/dL)	94 (69–148)	104 (72–148)	<0.0001
Glycemia (mg/dL)	86 (79–94)	83.5 (77–93)	<0.0001
SBP (mmHg)	110 (107–120)	120 (110–140)	0.006
DPB (mmHg)	70 (60–75)	80 (70–90)	<0.0001
Arterial hypertension, *n* (%)	31 (36.5)	8 (16)	0.011
Smoke *n* (%)	6 (7.1)	2 (4)	>0.05
IMT (cm)	0.8 (0.7–1)	0.6 (0.5–0.7)	<0.0001

*BMI* body mass index; *MetS* metabolic syndrome; *HDL* high-density lipoprotein; *LDL* low-density lipoprotein; *SBP* systolic blood pressure; *DBP* diastolic blood pressure; *IMT* intima-media thickness

Data of numerical and categorical variables are reported as median and interquartile range or number and percentage, respectively

In SLE patients, antibodies against double-stranded (ds) DNA were present in 35 (70%) patients and the prevalence of antiphospholipid antibody syndrome (APLS) was 20% (*n*=10). The median values of SLEDAI and SDI was, respectively, 8 (6–12) and 2 (1–3). SLEDAI [8 (6–12) vs 10 (6–13), *p*<0.01] and SDI [2 (1–3) vs 2 (0–3), *p*<0.001] were significantly higher in MetS patients than in patients without MetS.

In SSc patients, the median values of mRss, DAI, and DSS were 11 (6–17), 1.5 (0.7–3), and 5 (4–6), respectively. Antitopoisomerase I antibodies (Scl70) and anticentromere antibodies (ACA) were present in 33 (38.8%) and 35 (41.2%) SSc patients. The NVC showed early pattern in 14 (16.5%) SSc patients, active in 33 (38.8%) SSc patients and late in 38 (44.7%) in SSc patients. The prevalence of digital ulcers (DUs) was 56.5%. The median values of DAI, DSS, and mRss were 1.5 (0.7–3), 5 (4–6), and 11 (6–17), respectively. The median value of DSS was significantly higher (*p*<0.05) in MetS patients than in patients without MetS [7 (5–7) vs 5 (3–6)].

The median values of resistin and adiponectin were significantly (*p*<0.001) lower in SLE patients than in SSc patients (Table 2).

**Table 2:** Median value and interquartile range of serum level of resistin (ng/mL) and adineponectin (ng/mL) in systemic lupus erythematosus (SLE) patients and systemic sclerosis (SSc) patients with or without metabolic syndrome (MetS)

	SLE patients	SSc patients	*p* value
Resistin (ng/mL), median value	2.45 (1.47–3.8)	6.44 (1.53–7.98)	<0.001
Adineponectin (ng/mL), median value	7.64 (5.2–9.72)	9.94 (6.99–15.59	<0.001
	SLE patients with MetS	SLE patients without MetS	
Resistin (ng/mL), median value	4.01 (2.7–4.5)	1.92 (1.2–3)	<0.001
Adineponectin (ng/mL), median value	5.64 (4.96–8)	8.38 (6.54–11.01)	<0.05
	SSc patients with MetS	SSc patients without MetS	
Resistin (ng/mL), median value	2.71 (0.81–5.25)	6.48 (2.49–8.68)	>0.05
Adineponectin (ng/mL), median value	6.85 (3.36–13.55)	10.85 (7.68–15.59)	>0.05

In SLE patients with MetS, median values of resistin were significantly (*p*<0.001) higher than patients without MetS, conversely median values of adineponectin were significantly (*p*<0.05) lower in MetS than patients without MetS (Table 2).

In SSc patients, no differences of resistin and adiponectin were observed between patients with or without MetS (Table 2).

## Discussion

In this study, the prevalence of MetS was higher in SLE than in SSc patients. The median values of resistin and adiponectin were significantly lower in SLE patients than in SSc patients. In SLE patients with MetS, median values of resistin were significantly higher than patients without MetS, and conversely median values of adineponectin were significantly lower in MetS than patients without MetS. No difference of median value of resistin and adiponectin was observed in SSc patients with or without MetS.

Our results confirm the high prevalence of MetS in SLE patients with respect to SSc patients. SLE is often described as a disease that most often strikes reproductive-age women. This pattern is clearly seen in data for black women in the USA. However, in other populations, a different pattern is generally seen among women, with the highest age-specific incidence rates after age 40 years, as seen in a large study from the UK. The average onset of SSc occurs between 40 and 50 years. Less than 10 % of patients develop SSc before the age of 20. However, the incidence and prevalence of MetS increase with age [10, 14]. As SSc patients are older than SLE patients, we should expect a higher prevalence of MetS in SSc patients. We can suppose that the increased prevalence in SLE patients is due to other factors (inflammation, accelerated atherosclerosis) than age. SLE is characterized by accelerated atherosclerosis in which other non-traditional risk factors such as antiphospholipid and other autoantibodies, steroid therapy and systemic inflammation, are involved [30]. In SLE patients, lupus nephritis, active inflammatory disease, and SLE-related damage are risk factors for MetS development [31]. In our cohort, SLEDAI-2K and SDI are higher in SLE patients with MetS than SLE patients without MetS. The association of the MetS with inflammation is well known and the accumulation of adipocytes in the adipose tissue mass leads to the dysregulated production of adipokines and upregulation of pro-inflammatory cytokines [32]. Dysregulation of adipose tissue-derived bioactive molecules, termed adipokines, is recognized as common ground for MetS. However, adipokine dysregulation is paradoxically associated with lipodystrophy and aging. Centenarians, a model of healthy aging and longevity, are reported to exhibit preserved insulin sensitivity as well as favorable adipokine profiles, particularly high levels of circulating adiponectin. These observations suggest that adipose tissue excess as well as its aging is implicated in the regulation of adipokines, insulin sensitivity, and lifespan in humans. We can suppose that higher level of adipokines in SSc patients than SLE patients is due to age [33].

In our study, we found low level of adiponectin and resistin in SLE patients compared to SSc patients. We can suppose that the difference of serum concentration of adiponectin and resistin is due to different pathogenesis between SLE and SSc. In SLE patients, the hallmark is autoantibodies production and inflammation; conversely, endothelia damage and microvascular damage with fibrosis play a key role in the SSc pathogenesis [23, 24, 28].

In our SLE patients with MetS, serum levels of adiponectin are reduced; conversely, serum levels of resistin are increased. Concentrations of anti-inflammatory cytokines, ghrelin, adiponectin, and antioxidant factors were decreased in metabolic syndrome MetS [34]. Adipose tissue influences the immune system response, and the adipokines play a key role in SLE patients with MetS [18]. In SLE patients, the results of adiponectin serum level are discordant in the clinical studies. Several studies showed high levels of adipokine in SLE patients [35–37]. The discordance of reported serum level of adiponectin is due to presence of isoform of adiponectin with high molecular weight with proinflammatory activity or insulin resistance [30, 35]. It is well known that high adiponectin serum levels facilitate insulin sensitivity, while reduced adiponectin serum levels are associated with hypertension, dyslipidemia, and type 2 diabetes mellitus. It is still unclear whether the observed disequilibrium of adipokine systems in subjects with connective tissue diseases contributes to their development or only reflects the presence of inflammatory process [38].

In this study, we reported a low prevalence of MetS in SSc patients. In a small study, Peralta et al. found high prevalence of MetS (35%) in SSc, but the authors used different criteria of classification of MetS [16]. The median value of DSS was significantly higher in SSc patients with MetS than in patients without MetS. There are no studies on the MetS prevalence in SSc patients. We did not observe differences of serum levels of adiponectin and resistin between SSc patients with or without MetS. To our knowledge, there are no studies on the adipokine levels and MetS in SSc patients. Although the association between some autoimmune disease (SLE, RA) and accelerated atherosclerosis is well known, this is not well studied in SSc patients. Schiopu et al. demonstrate that subclinical atherosclerosis is significantly higher in patients with SSc compared with controls and that certain novel proteins (IL-2, keratinocyte growth factor, intercellular adhesion molecule 1, endoglin, plasminogen activator inhibitor 1, and insulin growth factor binding protein 3) are independently associated with vasculopathy and fibrosis process [39]. The antifibrotic effect of adiponectin is mediated by inhibiting TGF-β signaling, and low levels of adiponectin are detected in dcSSc sera, in fibrotic skin areas, and specimens of pulmonary fibrosis [40, 41]. Resistin is associated with vascular damage and fibrosis in SSc. Vasoconstriction and oxidative stress in the endothelial cells are characterized by high plasma levels of resistin. In addition, increased levels of resistin were found in SSc patients with pulmonary arterial hypertension and digital vasculopathy [19].

This study has several limitations. The selection by single center is a main limitation. The race or ethnicity influences the results of MetS prevalence and adipokines serum levels. In our study, all patients are Caucasian and the MetS syndrome and SLE is higher in black women. This could represent a bias of simple selection.

These results are of fundamental importance in the diagnosis of comorbidities, therapy, and prognosis. Since an accelerated atherosclerosis is present in SLE patients, it is necessary to evaluate the simultaneous presence of MetS to rapidly initiate a specific therapy that can slow down the progression of macrovascular damage of the disease and risk of CV events. In SSc patients, endothelial dysfunction and microvascular damage can be aggravated by the simultaneous presence of MetS. HMG-CoA reductase inhibitors, or statins, are potent plasma LDL-cholesterol (LDL-c) lowering agents. Statins play an important role to reduce atherosclerotic cardiovascular disease. Different statins have been administered in various experimental and clinical studies focused on autoimmunity. The results indicate that statins can modulate immune responses through mevalonate pathway-dependent and independent mechanisms. The anti-inflammatory and immune-modulating effects include cell adhesion, migration of antigen presenting cells, and differentiation, as well as activation, of T-cells [42].

In conclusion, prevalence of MetS is higher in SLE patients. In SLE patients, MetS showed an association with adipokine levels and inflammation/activity disease scores. In SSc patients, MetS was associated with severity of disease.
